# A Dual-Wavelength Fiber Laser Sensor with Temperature and Strain Discrimination

**DOI:** 10.3390/s22186888

**Published:** 2022-09-13

**Authors:** Arturo Sanchez-Gonzalez, Rosa Ana Perez-Herrera, Pablo Roldan-Varona, Miguel Duran-Escudero, Luis Rodriguez-Cobo, Jose Miguel Lopez-Higuera, Manuel Lopez-Amo

**Affiliations:** 1 Department of Electrical, Electronic and Communication Engineering, Public University of Navarra, 31006 Pamplona, Spain; 2 Institute of Smart Cities (ISC), Public University of Navarra, 31006 Pamplona, Spain; 3 Photonics Engineering Group, University of Cantabria, 39005 Santander, Spain; 4 CIBER-BBN, Instituto de Salud Carlos III, 28029 Madrid, Spain; 5 Instituto de Investigacion Sanitaria Valdecilla (IDIVAL), 39005 Cantabria, Spain

**Keywords:** artificial backscatter reflector, C-band, erbium-doped fiber laser, femtosecond laser, longitudinal mode behavior, multiparameter sensor, random fiber grating, simultaneous measurement

## Abstract

This work presents a dual-wavelength C-band erbium-doped fiber laser assisted by an artificial backscatter reflector. This fiber-based reflector, inscribed by femtosecond laser direct writing, was fabricated into a single mode fiber with a length of 32 mm. The dual-wavelength laser obtained, centered at 1527.7 nm and 1530.81 nm, showed an optical signal-to-noise ratio over 46 dB when pumped at 150 mW. Another feature of this laser was that the power difference between the two channels was just 0.02 dB, regardless of the pump power, resulting in a dual emission laser with high equalization. On the other hand, an output power level and a central wavelength instability as low as 0.3 dB and 0.01 nm were measured, in this order for both channels. Moreover, the threshold pump power was 40 mW. Finally, the performance of this dual-wavelength fiber laser enhanced with a random reflector for sensing applications was studied, achieving the simultaneous measurement of strain and temperature with sensitivities around 1 pm/με and 9.29 pm/°C, respectively.

## 1. Introduction

Multiwavelength fiber lasers are of great interest for telecommunications, terahertz wave generation, sensing, fiber-optic tests and measurement applications [1]. Erbium doped fibers are the main efficient and usual amplification medium in this kind of laser. However, Erbium-doped fiber is a homogeneous gain medium at room temperature, which leads to some secondary problems, such as a strong mode competition. This competition can cause power fluctuations higher than 1.5 dB [2], power flatness worse than 16 dB and, sometimes, the need for liquid nitrogen cooling to reduce the homogeneous gain broadening [3,4].

Among previous applications, the need for equalization in the emitted wavelengths of a fiber laser is especially important for telecommunications and sensing [5]. This equalization can be achieved by using complicated cavities and non-lineal effects [6], by using a wavelength-flat amplification media [7] or by using special gain flattening filters [8]. These methods complicate the laser structure, or increase the cost of the laser.

Raman amplification-based fiber lasers are a good alternative to achieve stable and equalized laser emissions, due to the inhomogeneous behavior. Furthermore, Raman-based random distributed fiber lasers, based on amplified Rayleigh backscattering, show outstanding stabilities, in single or multiwavelength configurations [9]. However, these lasers need high pump power of watts and cavities of some kilometers in length [10,11]. Therefore, they are not well suited to develop compact or low-cost equipment.

Femtosecond (fs) laser direct-write optical fiber structures have proven to be of great interest for a wide range of applications. In particular, the development of fiber-optic microstructures, based on refractive index modification under fs-laser irradiation, has resulted in different implementations in the field of optical fiber sensors: surrounding refractive index sensors [12], strain sensors [13], curvature sensors [14], or multiparameter sensors [15], among others. With this new fabrication technique, it is possible to desing optical fiber reflectors, with a tailored wavelength response, to achieve a flat and stable wavelength emission.

For instance, recently, a dual-wavelength fiber laser, based on an fs-laser direct-write random Bragg grating array, which showed the properties of the fs written reflector, has been reported [16]. That laser improved power stability over that of previous dual-wavelength single mode Er-doped fiber-based lasers [17].

Here, a new single-mode fiber (SMF) artificial backscatter reflector (ABR), especially designed to improve previously reported dual-wavelength lasers, is presented. With this reflector we have demonstrated a compact and simple structure. More important, this new reflector allows high-power stability, low threshold pump power and remarkable equalization with respect to previous Er-doped fiber dual-wavelength lasers, achieving, simultaneously, a high optical signal-to-noise ratio (OSNR). In terms of sensing capabilities, this newly designed fiber structure ensures simultaneous measurement of strain and temperature with sensitivities comparable to those of other classical reflectors.

## 2. Materials and Methods

### 2.1. Inscription Process

This SMF-based structure was inscribed following a process similar to the one presented in [18]. The random reflector was manufactured using a femtosecond commercial Fiber Laser Chirped Pulse Amplifier from CALMAR lasers, operating at 1030 nm wavelength, with 370 fs of pulse duration, and a variable pulse repetition rate available up to 120 kHz. Femtosecond laser processing provided an increase in the inhomogeneity of the fiber’s refractive index [19], resulting in an enhancement of the distributed dispersion. The SMF, located over a nano-resolution Aerotech stage motor, was placed on a slide and covered with a coverslip. Between them, an index-matching oil was deposited to limit fiber-induced aberrations [20]. Then, the laser pulses were tightly focused through a 100×/NA = 0.5 objective lens from Mitutoyo Corporation. Regarding the inscription parameters, a fast-changing pulse energy of 0.19 to 0.9 μJ, and a spatial period of 1 to 8, were selected for this ABR.

### 2.2. Reflector Characterization

**Figure 1:**
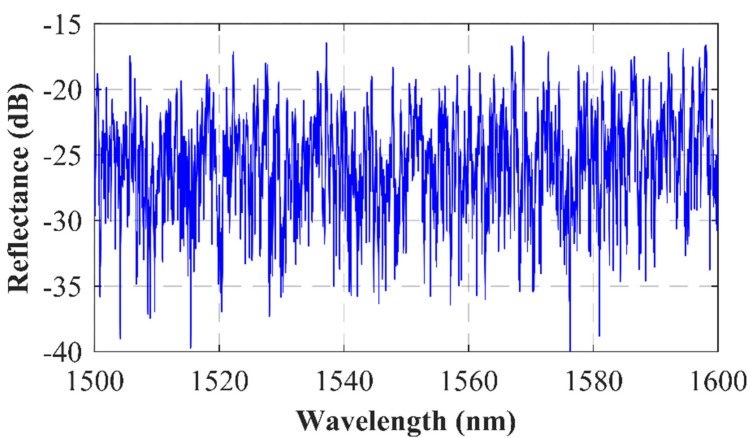
Return loss of the SMF–random reflector as a function of the wavelength measured by an OSA.

Firstly, the reflectance of this random reflector was characterized by means of a broadband light source and an optical spectrum analyzer (OSA; model MS9740A, from Anritsu). The results are shown in Figure 1, where it can be seen that the measured reflection spectrum presented a fairly flat response, so the emission wavelength of the generated lasers would be determined mainly by the gain shape of the erbium-doped fiber (EDF) employed, with its peak core absorption at 1531 nm. The combination of this slight return loss, associated with selected wavelength, and the EDF non-uniform gain, led to a two-wavelength stable emission, as seen below.

**Figure 2:**
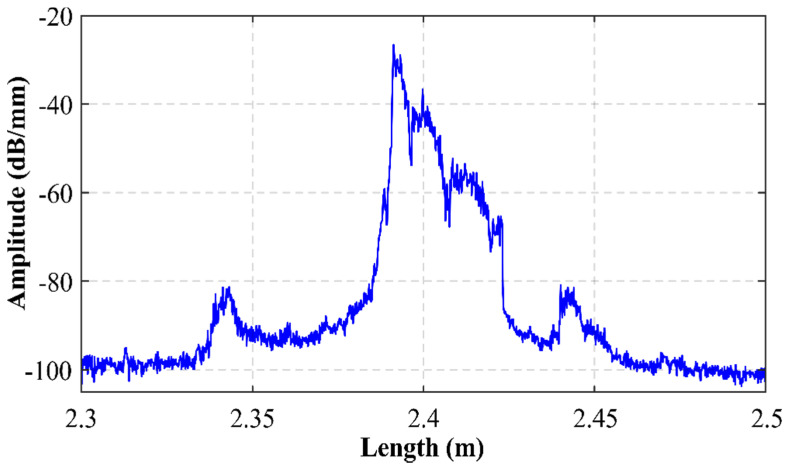
Backscattered optical power as a function of fiber length.

Secondly, an ultra-high spatial resolution optical backscattered reflectometer (OBR 4600, from LUNA), commonly used for fiber testing and sensing [21], was employed to retrieve the backscattered optical power, measured as a function of the length of this fiber-based random reflector inscribed into a single mode fiber. To avoid undesired reflections, free termination of the fiber-based reflector was immersed into index–matching oil. This measurement was performed in the time-domain acquisition mode, with a spatial resolution of 0.1 nm. As Figure 2 shows, the length of the inscription was around 34 mm and this fiber sample was located at 2.39 m from the OBR connector.

### 2.3. Experimental Setup

**Figure 3:**
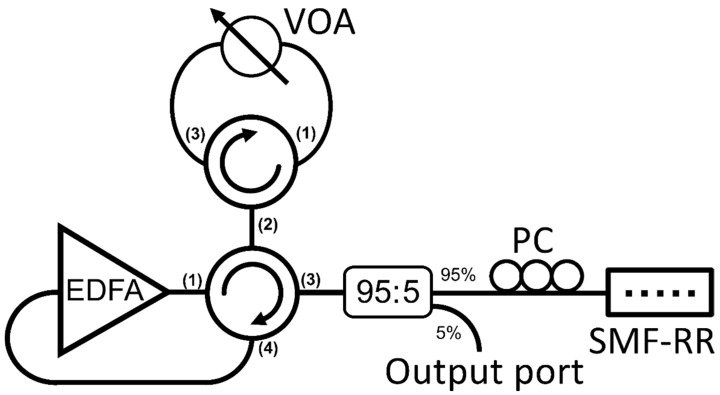
Experimental setup of the fiber laser resonator. SMF-RR: single mode fiber random reflector; PC: polarization controller; 95:5 optical coupler; EDFA: Erbium doped fiber optical amplifier; VOA: variable optical attenuator.

Figure 3 depicts the schematic diagram of the experimental setup carried out to evaluate the laser generation and sensor properties when using this single mode fiber random reflector (SMF–RR) within the resonator acting as a quasi-distributed mirror.

In this configuration, the light injected into the cavity ends at a fiber loop mirror (FLM) after passing through ports 1 and 2 of the 4-port optical circulator. The FLM includes a 3-port optical circulator, in which ports 1 and 3 are connected to a variable optical attenuator (VOA) to control the amount of light reflected into the rest of the cavity. After recirculating this signal, it reaches an optical coupler (OC). At this point, 5% of this signal is then extracted to be monitored by means of an optical spectrum analyzer, while the remaining 95% travels through a polarization controller (PC) to the random reflector, located at the right end of the cavity. This PC is also used to adjust the lasing stability. The reflected light from the random reflector finally recirculates via the PC, the OC, and ports 3 and 4 of the 4-port circulator, reaching the EDFA at its entry port and completing the round-trip through the cavity. The gain medium was 2.5 m of highly EDF I25 (980/125, Fibercore Inc., Las Vegas, NV, USA), suitable for C-band amplifiers with a core composition optimized for EDFAs in dense-WDM (DWDM) networks and a peak core absorption range from 7.7 to 9.4 dB/m at 1531 nm.

## 3. Results

### 3.1. Laser Performance

**Figure 4:**
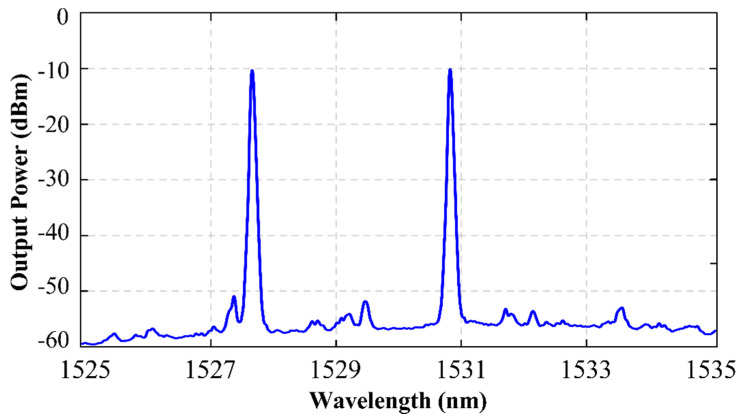
Output spectra of the highly equalized dual-wavelength C–band fiber laser assisted by a random reflector pumped at 150 mW and measured by an OSA.

**Figure 5:**
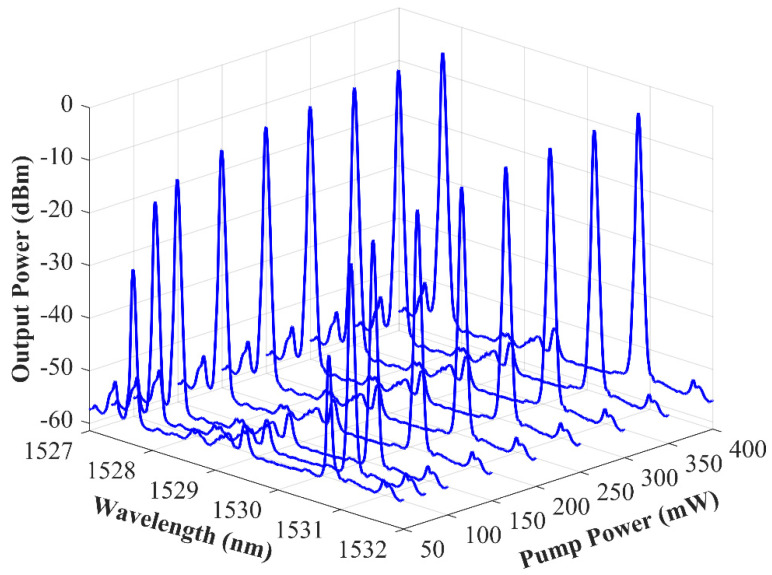
Output spectra of the highly equalized dual-wavelength C–band fiber laser assisted by a random reflector pumped from 40 to 400 mW and measured by an OSA.

The output spectrum of this erbium-doped fiber laser when pumped by a 976-nm source at 150 mW is shown in Figure 4. A dual-wavelength laser emission centered at 1527.7 nm and 1530.81 nm with an OSNR of 47.3 dB and 46.0 dB were measured, in that order. In addition, output power levels of −10.17 dBm and −10.15 dBm were achieved for each channel, respectively, showing a difference between them as low as 0.02 dB. This power difference between the two channels was kept reduced for all pumping power values, as Figure 5 illustrates, which makes this EDFL a highly equalized dual wavelength laser.

**Figure 6:**
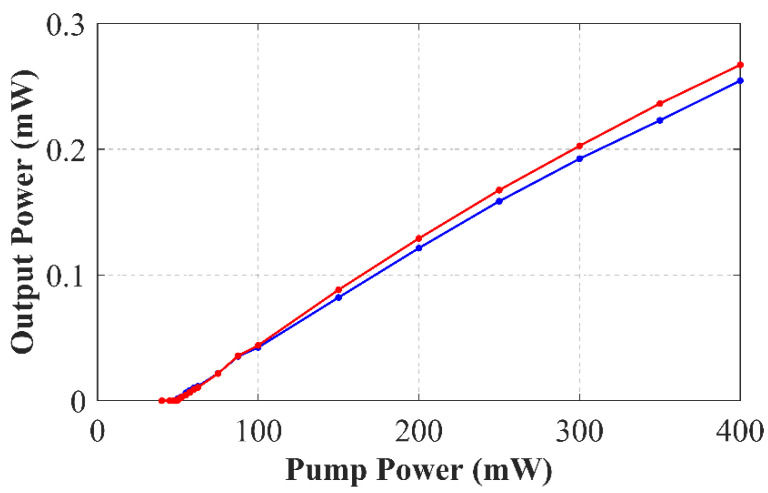
Relationship between the output power levels versus 976-nm pump power for the 1527.7 nm (blue line) and 1530.81 nm (red line) emission lines.

In order to evaluate the output spectra of this laser, the EDFL was pumped with powers ranging from 0 to 400 mW. Figure 6 depicts the relationship between the output power levels as a function of the 976-nm pump power for this SMF–RR–based fiber laser. This figure shows that both laser emission lines presented a threshold pump power of around 40 mW. A very high-power equalization for both emission channels could once again be observed in this new figure.

**Figure 7:**
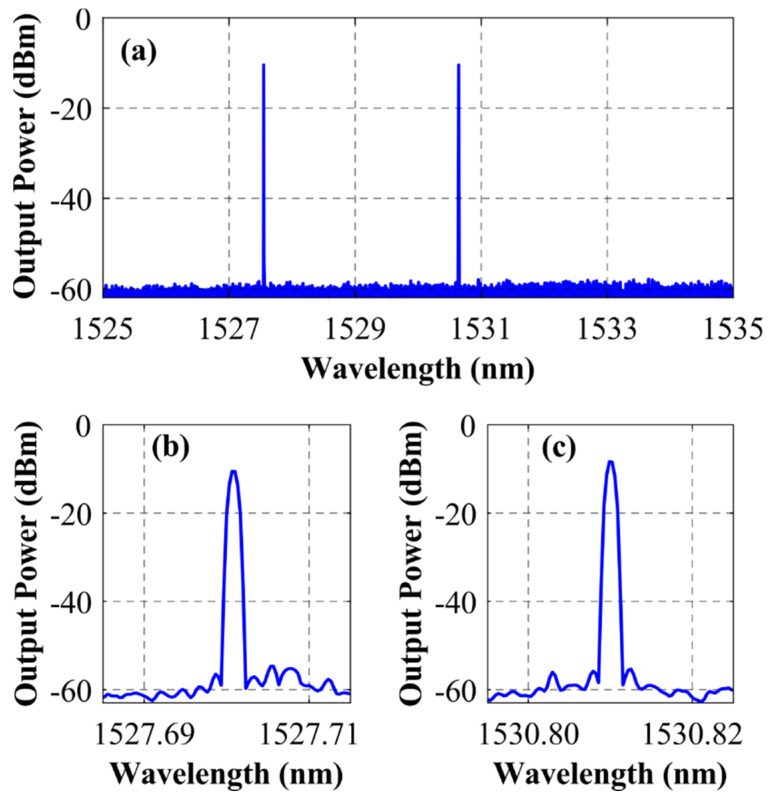
Output spectrum of the fiber laser when pumped at 150 mW and measured by a BOSA–C (**a**), with a detailed view of the 1527.7 nm (**b**) and 1530.81 nm (**c**) emission lines.

The longitudinal-modal behavior of this EDFL was also evaluated by means of a high-performance optical spectrum analyzer (Aragon Photonics BOSA–C). This device is capable of providing a spectral resolution of 0.08 pm with a dynamic range greater than 80 dB. Due to the fact that the total length of the cavity was short enough to fit the spectral resolution of the instrument, it was suitable to discriminate between longitudinal modes, thereby avoiding the need to analyze the electric beat with a tunable laser source (TLS). The result of this characterization is presented in Figure 7a where, taking into account the previous consideration, single-longitudinal mode (SLM) behavior can be verified. Detailed views of the output spectra of the 1527.7 nm and 1530.81 nm emission lines are shown in Figure 7b,c, respectively.

To investigate the response of each emission line in the absence of the remaining one, a narrowband (1.5 nm) bandpass tunable optical filter was introduced between OC and PC from Figure 3, centering its transmission band on each lasing wavelength one at a time. SLM operation persisted in both cases, discarding any notions that this behavior was due to mode competition and mode hopping annihilation by means of inter-channel seed light interaction [22]. Instead, the SLM lasing seemed to be exclusively due to the random nature of the reflector, as suggested by previous studies [23].

**Figure 8:**
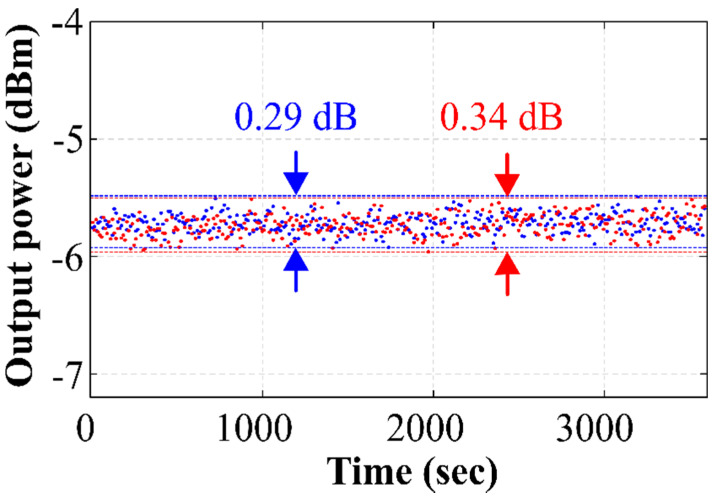
Output power stability of the 1527.70 nm (blue) and 1530.81 nm (red) emission lines when pumped at 400 mW, measured over 1 h with a CL of 95%.

The output power stability over time was analyzed. The peak powers of the 1527.7 nm and 1530.81 nm emission lines were tracked for different levels of pump power up to 400 mW, and for 1 h in each case. In every case, the power instability was found to be below 0.5 dB. Figure 8 shows the results achieved when pumped at 400 mW, reaching output power instabilities of 0.29 dB (blue lines) and 0.34 dB (red lines) for these two emission lines, with a confidence level (CL) of 95%. Due to the SLM behavior of the laser, these achieved stabilities were better than those reported for stable lasers in the introduction. Furthermore, we achieved SLM operation without the utilization of saturable absorbers, as needed in [24].

### 3.2. Sensor Performance

**Figure 9:**
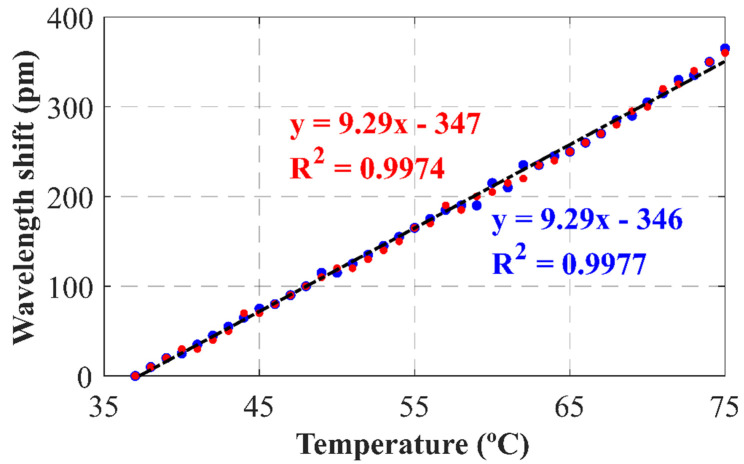
Wavelength shift as function of temperature when using the SMF−RR for the 1527.7 nm (blue) and 1530.81 nm (red) emission lines.

**Figure 10:**
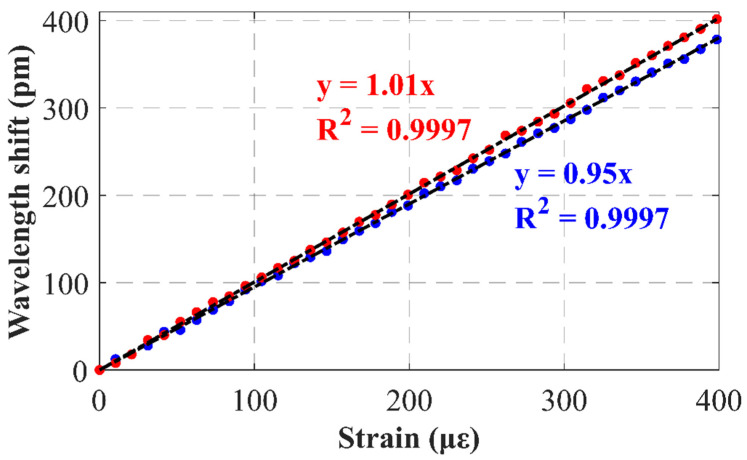
Wavelength shift as function of strain when using the SMF−RR for the 1527.7 nm (blue) and 1530.81 nm (red) emission lines.

Finally, the performance of this reflector as a temperature and strain sensor was experimentally evaluated. For this purpose, the random reflector was first placed into a climatic chamber while the resonance wavelength shift was measured, by means of the above mentioned OSA from Anritsu, as the temperature was steadily increased from room temperature to 75 °C. The results are presented in Figure 9 and, as can be seen, both emission lines presented the same strong linear trend, with an identical temperature sensitivity of 9.29 pm/°C. Secondly, the reflector was introduced into a custom-built motorized translation stage designed for strain measurement with a resolution of 0.09 με. As in the previous case, an OSA tracked the shift of both emission lines as strain was applied up to a value of 400 με, this time while temperature remained fixed. As a result, Figure 10 shows a strong linear dependency of the wavelength shift on the applied strain, reaching sensitivities of 0.95 and 1.01 pm/με for the 1527.70 and 1530.81 nm emission lines, respectively. These results remain comparable with those of commercial fiber Bragg Gratings (FBGs), with values around 13 pm/°C for temperature and 1.2 pm/με for strain [25].

The obtained strain sensitivities, although apparently similar, could be tested to be statistically different with a CL of over 99.99% by applying the hypothesis test for the slope of two regression lines [26]. By contrast, temperature sensitivities were to be considered equal at the same confidence level. Therefore, if the shift in wavelength for each emission line ($\Delta \lambda_{1}$, $\Delta \lambda_{2}$) was expressed as a function of the temperature variation (ΔT) and strain applied (Δε) in the usual way [27], the result obtained was a linearly independent system, which, in matrix form, could be written as follows:

$$\left(\begin{array}{c} \Delta \lambda_{1}\\ \Delta \lambda_{2} \end{array}\right)=\left(\begin{array}{cc} k_{T} & k_{\varepsilon_{1}}\\ k_{T} & k_{\varepsilon_{2}} \end{array}\right)\left(\begin{array}{c} \Delta T\\ \Delta \varepsilon \end{array}\right),$$

where $k_{T}$, $k_{\varepsilon_{1}}$ and $k_{\varepsilon_{2}}$ correspond to the temperature and strain sensitivities of 1527.70 and 1530.81 nm emission lines, respectively.

Accordingly, inverting the previous expression, the temperature variation and strain applied are given by:

$$\left(\begin{array}{c} \Delta T\\ \Delta \varepsilon \end{array}\right)={\left(\begin{array}{cc} k_{T} & k_{\varepsilon_{1}}\\ k_{T} & k_{\varepsilon_{2}} \end{array}\right)}^{-1}\left(\begin{array}{c} \Delta \lambda_{1}\\ \Delta \lambda_{2} \end{array}\right)=\frac{1}{k_{\varepsilon_{2}}-k_{\varepsilon_{1}}}\left(\begin{array}{cc} \frac{k_{\varepsilon_{2}}}{k_{T}} & \frac{-k_{\varepsilon_{1}}}{k_{T}}\\ -1 & 1 \end{array}\right)\left(\begin{array}{c} \Delta \lambda_{1}\\ \Delta \lambda_{2} \end{array}\right).$$

For the particular SMF-RR designed, using the previously measured sensitivities, temperature and strain applied simultaneously to the sensors could be deducted from the shift in laser emission lines wavelength as follows:

$$\left(\begin{array}{c} \Delta T\\ \Delta \varepsilon \end{array}\right)=\left(\begin{array}{cc} 1.873 & -1.765\\ -17.24 & 17.24 \end{array}\right)\left(\begin{array}{c} \Delta \lambda_{1}\\ \Delta \lambda_{2} \end{array}\right),$$

where wavelength shift is expressed in picometers, the temperature variation in Celsius degrees, and applied strain in micro-strain units.

These results underline the feasibility of employing random reflectors not only as mirrors, but also as substitutes for other classical temperature and strain sensors, as the necessity of temperature compensation in strain sensing applications is overridden by exploiting the different sensitivities of each emission line, while remaining comparable in magnitude to commercial FBGs.

## 4. Conclusions

In this work, a highly equalized dual-wavelength C-band fiber laser enhanced with a fiber random reflector was experimentally demonstrated. This random reflector was inscribed into a single mode fiber by femtosecond laser direct writing with a total length of 32 mm. Dual-wavelength, centered at 1527.7 nm and 1530.81 nm, with an optical signal-to-noise ratio of up to 47 dB, were obtained when pumped at 150 mW. These two laser emission lines had a very high level of equalization, as the difference between them was as low as 0.02 dB, independently of the pump power. In addition, both emission lines were shown to exhibit single-mode longitudinal behavior. These factors resulted in high stability in both output power levels and emission wavelength, varying by only 0.3 dB and 0.01 nm, respectively, over 1 h, and with a confidence level of 95%, when pumped at 400 mW. In addition to the above, the feasibility of these random reflectors not only as mirrors, but also as sensors, was experimentally demonstrated, reaching sensitivities of 9.29 pm/°C for temperature and around 1 pm/με for strain. In this regard, a laser capable of simultaneously measuring strain and temperature with sensitivities comparable with those of FBGs, while using a single quasi-point sensing element, was developed.

## Data Availability

Data underlying the results presented in this paper are not publicly available at this time but may be obtained from the authors upon reasonable request.
